# Product-Related Impurities in Clinical-Grade Recombinant AAV Vectors: Characterization and Risk Assessment

**DOI:** 10.3390/biomedicines2010080

**Published:** 2014-03-03

**Authors:** J. Fraser Wright

**Affiliations:** 1 Center for Cellular and Molecular Therapeutics, the Children’s Hospital of Philadelphia, ARC1216C, 3615 Civic Center Boulevard, Philadelphia, PA 19104, USA; E-Mail: wrightf@email.chop.edu; Tel.: +1-267-426-5801; Fax: +1-267-426-0363; 2 Department of Pathology and Laboratory Medicine, Perelman School of Medicine, University of Pennsylvania, ARC1216C, 3615 Civic Center Boulevard, Philadelphia, PA 19104, USA

**Keywords:** AAV vectors, impurities, clinical trials

## Abstract

Adeno-associated virus (AAV)-based vectors expressing therapeutic genes continue to demonstrate great promise for the treatment of a wide variety of diseases and together with other gene transfer vectors represent an emerging new therapeutic paradigm comparable in potential impact on human health to that achieved by recombinant proteins and vaccines. A challenge for the current pipeline of AAV-based investigational products as they advance through clinical development is the identification, characterization and lot-to-lot control of the process- and product-related impurities present in even highly purified preparations. Especially challenging are AAV vector product-related impurities that closely resemble the vector itself and are, in some cases, without clear precedent in established biotherapeutic products. The determination of acceptable levels of these impurities in vectors prepared for human clinical product development, with the goal of new product licensure, requires careful risk and feasibility assessment. This review focuses primarily on the AAV product-related impurities that have been described in vectors prepared for clinical development.

## 1. Introduction

Results in clinical trials continue to validate the great promise of adeno-associated virus (AAV)-based vectors for the delivery of therapeutic DNA to treat serious human diseases. In addition to the collective data documenting excellent safety and long-term gene expression in numerous animal models, clinical studies using AAV vectors for a variety of genetic diseases, including alpha-1-antitryspin deficiency, Batten’s disease, Canavan’s disease, cystic fibrosis, hemophilia B, Leber’s congenital amaurosis (LCA2), lipoprotein lipase deficiency, Pompe’s disease, Duchenne and limb girdle muscular dystrophies, and acquired disease, including Alzheimer’s disease, heart failure, Parkinson’s disease, rheumatoid arthritis and age-related macular degeneration, have been reported [1,2,3]. To date, one AAV-based product, Glybera (alipogene tiparovovec), for the treatment of lipoprotein lipase deficiency, has been licensed in the EU. The remarkable potential for long-term clinical benefit from a single administration of small amounts of recombinant AAV is supported by results emerging in recent human clinical trials. For example, in trials for LCA2, AAV-mediated delivery by sub-retinal injection of less than one microgram of DNA encoding retinal pigment epithelium-associated 65 kDa protein (RPE65) retinal isomerase achieved significant improvements in visual function for more than three years (and counting) [4,5,6,7,8,9,10]. For hemophilia B, AAV-mediated delivery to the liver of less than one milligram of DNA encoding human coagulation factor IX (FIX), a serine protease constituent of the coagulation cascade, achieved continuous therapeutic levels of FIX for more than two years (and counting) [11], corresponding to *in vivo* production of an estimated 200 milligrams of the therapeutic transgene product FIX per year per patient. While challenges remain, the results achieved so far in human clinical trials and the licensure of the first AAV product bear out the therapeutic and commercial promise of AAV-mediated therapeutic gene delivery. The expanding clinical pipeline and advancing stage of development of recombinant viral vectors for therapeutic gene transfer emphasize the need to ensure that products based on this new therapeutic platform are characterized and manufactured to rigorous and validated tolerances of purity, potency and safety. 

A discussion of the purity, homogeneity and impurity profile of a parenteral therapeutic product should consider the complexity of that product. While less complex small molecule drugs demonstrate relatively higher purity and homogeneity, more complex biotherapeutics, such as monoclonal antibodies, show greater product heterogeneity and higher levels and diversity of residual impurities that must be adequately defined and controlled. AAV vectors are yet more complex; the desired AAV vector is composed of an icosahedral assembly of 60 capsid proteins (VP1, -2, -3) assembled together in a defined stoichiometry [12], together with a single-stranded DNA molecule encoding the therapeutic expression cassette encapsidated within the AAV particle. The subcomponent multiplicity of recombinant AAV correlates with a higher potential for the formation of product-related impurities. As one relevant example, residual baculovirus genetic sequences unintentionally encapsidated in AAV1 capsids and co-purified with the product was one of six major product quality objections raised during the licensure assessment of Glybera by the European Medicine Agency [13]. For this review article, otherwise normal AAV capsid particles that are empty or that encapsidate fragments of nucleic acids other than the intended therapeutic expression cassette are examples of vector product-related impurities. A relevant feature of AAV product-related impurities is that they closely resemble the desired AAV vector, presenting challenges for the manufacturing process, including the need to optimize upstream (cell culture) processes to reduce their biosynthetic generation, and downstream (purification) processes to reduce or remove these moieties that may closely resemble the desired product. While analytical methods previously developed and validated for licensed biologics are useful and applicable to AAV vector manufacturing process-related impurities, such as residual production (host) cell proteins and nuclease-sensitive nucleic acids, new analytical methods have been required to characterize and quantify vector product-related impurities in recombinant AAV investigational products. Some new methods are not yet validated to the standards required for licensed products, and additional manufacturing experience and analytical method development are needed. This review aims to provide a general summary of the types of impurities that should be considered in the context of development of AAV-based clinical products, focusing on the characteristics of vector product-related impurities, risk assessment and how they can be reduced by manufacturing process optimization. 

## 2. Types of Impurities

The term “impurity” as used herein is defined as any component present in purified AAV vectors that is not the desired product, a product-related substance (*i.e.*, a molecular variant that exhibits potency and safety comparable to the desired product) or an intended formulation excipient [14]. The term impurity here will also include helper-virus-dependent replication-competent AAV particles (rcAAV), which can be unintentionally generated by recombination events in the biosynthetic milieu of the vector generation system. This article will not address contaminants, which are adventitious agents (e.g., microbial species) unintentionally introduced during the manufacturing process of the drug product. In contrast to impurities, which are expected to be present, but should be characterized and substantially reduced and controlled to an acceptable range, contaminants should be strictly avoided by adherence to good manufacturing practices (GMP), including the adequate qualification of chemical and biological raw materials and performance of vector generation and purification steps using aseptic techniques [15,16,17,18,19].

**Figure 1:**
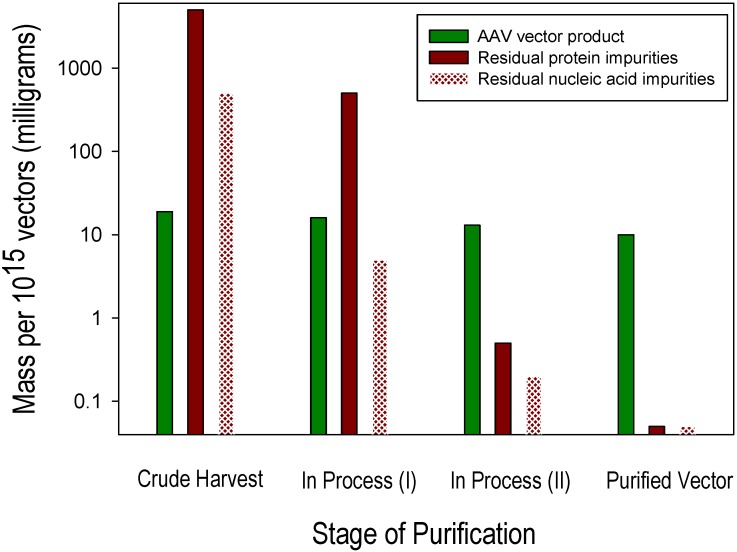
Adeno-associated virus (AAV) vector and impurity levels during purification.

Impurities remaining after vector purification include residual levels of proteins and nucleic acids that derive from the components of the cell culture system within which the vector product is generated. Examples of two abundant sources of protein impurities are residual proteins derived from the production cells (host cell proteins) and from bovine serum (if used), such as residual bovine serum albumin. Similarly, two abundant sources of nucleic acid impurities are residual nucleic acid constituents of the production cells (host cell DNA and RNA) and DNA from helper components (e.g., plasmids or viruses) added to support vector production. These biological components of cell culture-based production systems are highly abundant and diverse prior to purification, and their substantial removal from the small relative mass of the vector generated is a critical objective for AAV vector purification process development and optimization. Residual host cell DNA is present in two forms: (1) as a nuclease-sensitive process-related impurity, *i.e.*, non-specifically co-purified with the desired AAV vector product; and (2) as a nuclease-resistant product-related impurity, *i.e.*, encapsidated within AAV particles. Minimizing these distinct forms of residual host cell DNA requires different manufacturing process optimization strategies. A model of the purification power and specificity that is required to purify AAV vectors generated in cell culture using helper virus-free transient transfection of Human Embryonic Kidney 293 (HEK293) cells is provided in the following example in which the objective is the recovery of 10^15^ highly purified recombinant AAV vectors, *i.e.*, DNAse-resistant vector genome particles as measured by qPCR and substantially free of process-and product-related impurities. This example “lot” size corresponds to that required to support a Phase 1 clinical trial for many diseases. Approximately 10 mg of an AAV vector drug substance (10^15^ vector particles containing ~6.5 mg of AAV capsid protein and ~3.5 mg of vector DNA) must be purified from a cell culture milieu containing ~4 g of non-vector protein (3 g of HEK293 cellular protein from ~10^1^^0^ cells and ~1 g of fetal bovine serum protein) and ~350 mg of non-vector nucleic acids (320 mg of HEK293 cellular nucleic acids and 30 mg of production plasmid DNA). Figure 1 shows approximate quantities of vector and impurities at the pre-purification (crude harvest) stage, two sequential intermediate in-process stages of purification and after completion of purification. In order to reduce the initially abundant and diverse protein and nucleic acid impurities to a level corresponding to ≤2% of the desired vector product, they must be selectively reduced approximately 10^5^- and 10^4^-fold, respectively, by a process that also achieves an acceptable (e.g., ≥50%) yield of the desired AAV vector. 

As a preliminary guideline, purity specifications can be based on those developed and established for licensed biologic products, such as recombinant proteins and vaccines [20]. Since AAV vectors under development as drug products, unlike most virus-based products that have been developed and licensed as vaccines designed to stimulate immune responses, aim to establish durable gene expression, their design and methods used for their preparation should aim to minimize the potential for unintended immune responses. Hence, impurities that have the potential to contribute to immune responses should be identified and minimized. The spectrum of residual impurities in purified recombinant AAV reflects the nature of the manufacturing of the raw materials used and the details of the manufacturing process. For example, AAV vector production systems based on human cell lines [21,22,23,24] and insect cell lines [25,26,27] result in qualitative differences in the nature of residual host cell proteins and nucleic acids, with corresponding differences in the risk of potential immunotoxicity in human subjects, even when the quantities of impurities are comparable. Since process-related impurities in AAV vectors are similar in nature, risk profile and manufacturing process optimization requirements to established biologics [20,28,29], they are summarized in Table 1 and discussed only briefly. The more unique and challenging AAV vector product-related impurities are summarized in Table 2 and discussed in detail. 

## 3. Process-Related Impurities in AAV Vectors

Process-related impurities are derived from the manufacturing process of the raw materials and components, including cell substrate, cell culture medium, helper components, such as viruses and plasmid DNA, and purification-related process components, but are not structurally related to the product. A list of process-related impurities pertinent for AAV vector manufacturing, the suggested methods for their measurement and the type of toxicity that they may cause are provided in Table 1.

**Table 1:** Process-related impurities encountered in AAV vector manufacturing.

Process-related impurity	Method of measurement	Potential toxicity
Residual host cell DNA/RNA (nuclease-sensitive)	qPCR using amplicons to generic host cell genome (e.g., *18SRNA* gene)	Genotoxicity
	qPCR using amplicons for sequences of specific concern (e.g., *AdE1*)	
Residual host cell protein	ELISA using polyclonal antibodies detecting representative proteins [30]	Immunotoxicity
Residual plasmid DNA (nuclease-sensitive)	qPCR using amplicons for non-vector genome sequences	Genotoxicity
Residual helper viruses (nucleic acids and proteins)	qPCR using amplicons for helper virus sequences	Immunotoxicity, Genotoxicity, Infectious risk
	Infectious titer of helper viruses;	
	ELISA or Western blotting for helper virus proteins	
Residual cell culture medium components, antibiotics, supplements, inducers, *etc.*	Various, depending on component	Various
Residual purification buffers, chromatography media ligands, centrifugation media, detergents, enzymes, inorganic salts, *etc.*	Various, depending on component	Various

## 4. Product-Related Impurities in AAV Vectors

Vector product-related impurities are structurally related to, but distinct and not comparable to, the desired vector product with respect to efficacy and safety. Such impurities can include biosynthetic intermediates, particles of incorrect composition (e.g., nuclease resistant nucleic acid impurities packaged in AAV capsid particles) and degraded, oxidized and aggregated forms of the vector product. Product-related substances are molecular variants of the product, formed during manufacturing and/or storage that are comparable to the product with respect to efficacy and safety. AAV product-related substances may include individual particles with minor variation in the stoichiometry of VP1, -2 and -3 that still retain infectivity or limited heterogeneity in the 5' ends of expression cassettes packaged within AAV [31]. In contrast, AAV product-related impurities are inactive or otherwise not comparable in efficacy to the desired vector product and may represent safety concerns. Product-related impurities that have been described for AAV vectors, including suggested methods for their characterization and potential toxicity, are provided in Table 2.

**Table 2:** Product-related impurities encountered in AAV vector manufacturing.

Product related impurity	Method for measurement	Potential toxicity
AAV empty capsids	Ultracentrifugation; electron microscopy; spectrophotometry [32]; ion exchange chromatography [33]	Immunotoxicity
Encapsidated host cell nucleic acids (nuclease-resistant)	qPCR using amplicons to generic host cell genome sequences	Genotoxicity, Immunotoxicity
	qPCR using amplicons to specific sequences of concern (e.g., *E1A*)	
Encapsidated helper component DNA (nuclease-resistant)	qPCR using amplicons for helper backbone sequences	Genotoxicity, Immunotoxicity
Replication-competent AAV	Ad-dependent amplification	Immunotoxicity
Noninfectious AAV particles	Ad-dependent infectivity in susceptible cells	Immunotoxicity
Other, including aggregated, degraded, and oxidized AAV vectors	Various, including size exclusion chromatography; dynamic light scattering; electrophoresis, *etc.*	Immunotoxicity

### 4.1. AAV Empty Capsids

#### 4.1.1. Description

AAV empty capsids are composed of an AAV capsid shell essentially similar to that of the desired product, but lacking a nucleic acid molecule packaged within. AAV empty particles characterized by density centrifugation and electron microscopy methods may include particles containing small fragments of DNA [31] that are not readily distinguished from completely empty capsids. Empty capsids are known to be generated in high levels in several AAV vector production systems, including transient transfection of HEK293 cells, in which empty capsids can correspond to 50%–90% of the total AAV particles generated in cell culture [32,34]. Purification of AAV vectors by methods that do not substantially remove this product-related impurity can result in final vector preparations that contain excess (e.g., 10-fold) empty capsid particles relative to the desired vector product. Even within one vector production method, empty capsid content relative to the vector can exhibit high lot-to-lot variability reflected in the purified product if empty capsids are not removed or otherwise controlled [32]. 

#### 4.1.2. Risk Assessment

A concern with having large amounts of empty capsids in clinical preparations of AAV vectors is their potential to exacerbate adaptive immune responses directed to the viral capsid antigen. A cluster of differentiation (CD) 8^+^ T-cell response directed against AAV capsid was implicated in the recognition and clearance of transduced hepatocytes in clinical trials using AAV2-hFIX [35,36] and AAV8-hFIX [11], and peptides derived from empty capsids themselves have been demonstrated to gain access to MHC Class I presentation pathways [37]. Hence, substantial separation of empty capsids is supported as a prudent strategy to minimize the potential for immunotoxicity associated with viral antigen in a therapeutic vector dose. 

A second concern with including a large excess of AAV empty capsids is that they may reduce the transduction of target cells by competing for vector binding sites on those cells, an effect that could increase vector dosing requirements. Parker and colleagues reported that transgene expression was inhibited by the presence of empty capsids following high dose (>10^13^ vector genome (vg)/kg) administration of AAV2 vectors to mouse liver [38]. This competitive inhibitory effect may be tissue- and route of administration-specific, with the potential for the inhibition of transduction likely to be greater when the vector is directly administered to tissues, such as brain parenchymal and the subretinal layers in the eye, for which the multiplicity of infection (MOI) for vector transduction is higher (range: 10^5^–10^6^ vg per cell); and of less concern for systemic administration of AAV targeting the liver for which MOI is estimated to be lower (range: 10^3^–10^4^ vg per cell). 

However, empty capsids have also been reported to have a beneficial effect under certain conditions. Based on their immunological similarity, empty capsids can act as effective decoys to reduce the neutralization of AAV vectors by pre-existing antibodies and thereby increasing the target tissue transduction following systemic administration [39]. This potential benefit of antibody decoys should be balanced against the risk of exacerbating deleterious T-cell responses and competitive inhibition of vector transduction of target cells, on a clinical application-specific basis. 

#### 4.1.3. Process Optimization

Given the structural similarity between AAV empty capsids and the desired vector product, the removal of empty capsids is challenging. Furthermore, purification processes that fail to separate the vector and empty capsids are predicted to also fail to separate other vector-related impurities, such as AAV particles containing fragments of the host cell and helper component DNA, described below. Two general approaches to reduce empty capsids in AAV vector preparations are: (1) the optimization of the components used and the conditions of vector generation in cell culture to improve vector packaging efficiency and reduce the proportion of empty-to-vector-containing AAV particles; and (2) the optimization of the purification process steps to substantially reduced or removal of empty capsids during vector purification. For the latter approach, gradient ultracentrifugation using cesium chloride [40,41] or iodixanol [42] is effective, but presents challenges for scalability. Empty capsids are also amenable to separation from the vector by ion exchange chromatography, as reported for AAV2, AAV6 [43] and AAV5 [26,44] vectors.

### 4.2. Residual Host Cell Nucleic Acids Packaged within AAV Capsids

#### 4.2.1. Description

AAV-encapsidated host cell DNA impurities correspond to heterogeneous fragments of host cellular DNA unintentionally packaged within AAV capsid particles. The packaging of fragments of mammalian producer cell genomic DNA has been reported to be generated at a frequency of 1% to 3% of the AAV genome-containing particles, as inferred from the levels of this product-related impurity observed in purified AAV vector particles [45]. These heterogeneous nucleic acids from the host cell are present in excess relative to vector genome DNA templates in the cell culture milieu in which AAV vectors are generated. Hence, even inefficient packaging of host cell DNA into AAV particles can result in formation of a significant amount of this product-related impurity during AAV vector generation. The mechanism of the packaging of fragments of host cell DNA within AAV particles is not understood. The assembly of AAV capsid particles and the packaging of wild-type AAV genomes within these particles (encapsidation) has been described to occur in the nucleus of infected cells [46,47], and the packaging of recombinant AAV vector genomes is predicted to similarly occur in the nucleus. The packaging of the vector genome is thought to be mediated by the AAV Rep protein that bridges the Rep binding element (RBE) located on the vector genome-associated Inverted Terminal Repeats (ITRs) to sites proximal to pores located at the five-fold axis of symmetry on the surface of preformed AAV empty capsids, mediating the translocation of single-stranded DNA vector genomes into the preformed capsids. Whether such a Rep-mediated mechanism might operate in the packaging of single-stranded DNA fragments from the host production cell genome is speculative. However, the rescue of wild-type AAV after natural latent infection, which involves the recovery of the AAV genome from the AAV S1 genome integration site, supports the feasibility for such a mechanism. Since AAV ITRs are not expected to be present in the production cell genome, imperfect fidelity of the ITR-specific mechanism, perhaps exacerbated by the presence of excess helper virus gene products, might involve the binding of Rep to motifs in the host cell genome that share homology with the ITR RBE (RBE-like motifs) and thereby inadvertently acting as weak packaging signals. If RPE-like motifs occur throughout the genome of production cells, this could account for the encapsidation of heterogeneous host cell DNA fragments in AAV particles.

#### 4.2.2. Risk Assessment

Depending on the origin of the cells used for AAV generation, *i.e.*, human or non-human origin, genotoxicity and immunotoxicity are two theoretical risks. The presence of transforming factors, including the adenovirus *E1* gene in HEK293 cells and human papillomavirus *E6* and *E7* oncogenes in HeLa cells, as well as the potential presence of other activated oncogenes in various vector production cell substrate genomes, raises concerns over the presence and quantity of these potentially deleterious DNA sequences in viral vector preparations [48,49,50]. The concern that such residual DNA impurities might express oncogenes led to existing guidelines that residual DNA amount and size be controlled; residual cell-substrate DNA should be ≤10 ng per dose, with a median DNA size of 200 bp or lower [19,51]. These guidelines can be addressed by established process optimization strategies when residual host cell DNA is present as a nuclease-sensitive process-related impurity; however, residual nuclease-resistant host cell DNA packaged within AAV capsids is uniquely challenging [52]. As a semi-quantitative illustration, assuming a highly efficient purification process that effectively purifies AAV icosahedral particles containing vector genomes and only those packaged nucleic acid fragments corresponding to the mass of the vector genome, ~1% of these particles are still predicted to contain heterogeneous fragments of host cell DNA [45]. In this case, the World Health Organization (WHO) guideline for residual host cell DNA quantity cannot be met for a vector dose exceeding 2 × 10^11^ vg. Furthermore, the residual host cell DNA impurity is predicted to be present as single-stranded DNA fragments of ~4700 nt, which exceed the World Health Organization guideline for size. A mitigating feature of residual host cell DNA fragments packaged by AAV vectors is its predicted single-stranded nature, rendering it unstable and likely to be degraded quickly following unpackaging in the nuclei of transduced cells. Assuming the encapsidated residual host cell DNA represents AAV genome-sized fragments distributed randomly throughout the ~3 × 10^9^ nucleotide genome of human production cells, the predicted frequency of the packaging of any specific sequence within the host cell genome is ~1 copy per 10^8^ vector genome particles. While AAV encapsidated host cell DNA falls under the existing regulatory guidelines regarding amount per dose and average size, these limits may require re-consideration for gene therapy products based on the unique characteristics of this vector product-related impurity. 

The qualitative nature and risk profile of AAV packaged residual host cell DNA can be influenced by the choice of the production the cell system used. AAV vectors can be produced in human cell lines [21,22,23,24] or insect cell lines [25,26,27]. Relative to the use of human cell lines that will result in residual human genomic DNA packaged within the AAV vector product, perhaps representing a greater risk of genotoxicity, due to the potential for homologous recombination with genomic sequences in transduced human cells, the use of insect cells resulting in packaged insect cell genomic DNA is predicted to reduce this genotoxic risk, but increase the risk of immunotoxicity by unintended expression of insect cell polypeptides in transduced tissues. 

#### 4.2.3. Process Optimization

Based on close similarity with the desired vector product, it is difficult to eliminate AAV packaged host cell DNA impurities by vector purification methods. The separation of AAV particles based on their density by gradient centrifugation can at least remove AAV packaged nucleic acid impurities that differ significantly in length from the vector genome based on the different density of the respective particles. The purification of AAV vectors by isopycnic gradient ultracentrifugation as a supplement to chromatographic purification steps reduced encapsidated residual HEK293 DNA 4.7-fold [45], but may represent a scalability challenge for large clinical programs. The development of AAV capsid variants with higher transduction efficiency could further lower these impurities on a per-dose basis by reducing the vector genome particle dose required for efficacy.

### 4.3. Residual Helper DNA Sequences Packaged in AAV Capsids

#### 4.3.1. Description

Similar to encapsidated host cell DNA, the packaging of helper DNA sequences corresponding to fragments of DNA derived from helper plasmids or viruses within AAV capsids has been described. Packaged residual DNA impurities derived from vector template and helper sequences were reported to range from 1% to 8% of vector genome DNA in purified vector particles [45,53,54]. Residual nuclease-resistant recombinant baculovirus DNA was described as an impurity and product quality concern during the licensure assessment for Glybera [13]. The packaging of helper component DNA sequences within AAV particles may involve a mechanism analogous to that proposed above for host cell DNA, *i.e.*, recognition by the AAV Rep protein of RBE-like motifs in helper component DNA sequences. This mechanism may account for low levels of heterogeneous fragments from various helper component DNA sequences other than the AAV ITR-containing expression cassette sequences. However, the more abundant and homogeneous form of this vector product-related impurity has been reported to be derived from the ITR-containing vector template that is present and required in all vector production systems [13,45,53,54]. The presence of AAV ITRs flanking the vector genome expression cassette provides a *bona fide* Rep binding element, but in reverse orientation for the packaging of backbone sequences adjacent to the expression cassette. To account for the relative abundance of ITR proximal sequences in encapsidated helper component sequences, a small, but significant frequency of such reverse packaging appears to occur, resulting in the packaging of the ITR contiguous plasmid or bacmid backbone sequences adjacent to the expression cassette. 

#### 4.3.2. Risk Assessment

A primary concern of residual helper DNA sequences packaged within AAV particles is the possibility of the unintended expression of immunogenic peptides. DNA fragments postulated to occur herein as a result of promiscuous Rep binding to RBE-like motifs in helper component sequences are predicted to be heterogeneous and single stranded, with a low probability of being stabilized by annealing to homologous sequences and likely to be readily degraded following unpackaging in vector transduced cells. In support of this hypothesis, one study reported that residual cap DNA impurities in highly purified AAV2 vectors were not transcribed [45]. However, ITR-associated DNA fragments arising from the postulated reverse packaging from ITRs on the therapeutic expression cassette-containing helper sequences are predicted to be more homogeneous and more likely to be stabilized after packaging by the initiation of second strand synthesis and/or by annealing to homologous sequences. If AAV encapsidated, ITR-linked backbone sequences were present in sufficient quantities in a preparation of an AAV vector and if such fragments inadvertently contained weak promoters and transcriptional start sites, there is more risk for immunotoxicity. One of the six major product quality objections raised during the evaluation of Glybera was the possibility of intact open reading frames within residual encapsidated baculovirus DNA and the associated potential for baculovirus protein expression in cells transduced by the product [13]. Further concerns include the potential to transfer prokaryotic sequences, such as antibiotic resistance genes [53], which can be mitigated by employing resistance genes to antibiotics not commonly used in clinical practice. 

#### 4.3.3. Process Optimization

The use of an “oversized” backbone in the vector plasmid used for helper virus-free AAV production in HEK293 cells was reported to reduce residual plasmid backbone packaging ~5-fold, and the purification of AAV vectors by isopycnic gradient ultracentrifugation in addition to ion exchange chromatography further reduced encapsidated residual plasmid DNA 2.8-fold (*p* = 0.010) [45]. The removal of encapsidated DNA impurities of sizes similar to the actual vector genome remains a fundamental challenge, and a certain level of this impurity cannot be further reduced by known purification methods. However, the risk associated with the expression of unintended open reading frames proximal to the expression cassette ITRs on helper components can be reduced by ensuring that all nucleotide sequences in the helper backbone sequences proximal to AAV ITRs lack sequences with the potential to act as promoters or sites of the initiation of transcription.

### 4.4. Replication-Competent AAV Species

#### 4.4.1. Description

Helper virus-dependent replication-competent AAV (rcAAV), also referred to as “wild-type” or “pseudo-wild-type” AAV, is an AAV capsid particle containing AAV *rep* and *cap* flanked by ITRs that is able to replicate in the presence of a helper virus. The generation of rcAAV during vector production has been described to involve homologous or non-homologous recombination events between AAV ITRs present on the vector expression cassette production template with the *rep* and *cap* sequences that are present in vector packaging helper components [55,56,57]. Vector production systems currently in use have been optimized to reduce rcAAV to levels ≤1 rcAAV per 10^8^ vector genomes. This value is in part a function of the limit of sensitivity of analytical methods for rcAAV, which typically detect the amplification of AAV *rep* and/or *cap* DNA following multiple rounds of passaging of a recombinant AAV test article on a permissive cell line in the presence of adenovirus. 

#### 4.4.2. Risk Assessment

Infection by wild-type AAV Type 2 is common in the human population [58], with no known associated pathology. Furthermore, wild-type AAV is unable to replicate autonomously and requires co-infection with helper viruses, such as adenovirus and herpesvirus that, in contrast to wild-type AAV, do cause pathology in humans. Therefore, the adventitious exposure to helper viruses required for the replication of any residual rcAAV present as a product-related impurity in an AAV vector administered to a human subject, not the rcAAV itself, would be the primary infectious toxicity concern in a human subject that received the AAV vector. However, the expression of AAV *rep* or *cap* from rcAAV present in an AAV vector investigational product increases the risk of immunotoxicity in vector-transduced tissues. In the absence of rcAAV replication, it seems likely that rcAAV would need to be present as a significant fraction (e.g., >0.1%) of *bona fide* AAV vectors to infect a significant proportion of vector transduced cells and render them susceptible to direct recognition by capsid specific CD8^+^ T-cells. However, it is feasible that trace amounts of rcAAV could help trigger the formation of such CD8^+^ T-cells, which could then recognize vector-transduced cells presenting peptides derived from the input capsid protein component of the vector inoculum by a cross-priming mechanism [59]. The risk of contributing to immunotoxicity through mechanisms that remain incompletely understood supports the importance of the reduction of rcAAV to the lowest levels achievable in AAV vectors prepared for clinical investigational product development. 

#### 4.4.3. Process Optimization

Helper virus-dependent rcAAV closely resembles authentic AAV vectors, and these entities cannot be separated by purification process steps. Rather, strategies to minimize rcAAV formation during vector generation in cell culture are required. Approaches that have been reported include elimination of overlapping sequences in production components to prevent rcAAV generation by homologous recombination [55], inactivation or replacement of the p5 promoter region implicated in recombination events [60], separation of AAV rep and cap sequences by providing them on separate helper plasmids [56], incorporation of a microRNA binding cassette proximal to the AAV cap helper component [61] and sequestration of AAV helper *rep* and *cap* from vector genome DNA components in different sub-cellular compartments [62].

### 4.5. Non-Infectious AAV Vector Particles

#### 4.5.1. Description

Infectious titers and particle-to-infectivity (P:I) ratios are classical virology methods applied to the characterization of recombinant AAV vectors as measures of functional activity. For recombinant AAV, non-infectious particles in a broad sense include many of the product-related impurities described above, e.g., empty AAV capsids, and non-replication competent encapsidated host cell and helper nucleic acids. Herein, a narrower meaning of non-infectious AAV vectors will be used; namely, AAV capsids that contain the intended vector genome, but that do not demonstrate replication using available cell-based assays in the presence of helper sequences. It is especially challenging to measure the infectivity titer of AAV vectors, because: (1) the vector is by design not able to replicate in transduced cells (even the parent virus is unable to replicate in the absence of helper virus), and therefore, multiple helper virus genes, as well as the wild-type AAV rep must be provided to any cell culture system designed to detect infectivity as measured by AAV genome replication; (2) cytopathic effects (CPE) caused by AAV vector infection are weak and, if present, are unlikely to be discernable above the strong CPE caused by helper viruses that must be added to an assay for AAV vector infectivity; and (3) most available cell lines do not support efficient infection by vectors based on AAV serotypes other than AAV2, even when sufficient helper virus genes are provided. Hence, P:I ratios measured using existing methods do not provide accurate values, especially for non-AAV2 vectors. For AAV2 vectors, P:I ratios in the range of 5–1000 have been reported with permissive cell culture systems using adenovirus to provide helper virus functions [42,63,64]. Infectivity assays using recombinant herpesviruses to provide helper functions have been developed and used successfully for other serotypes reporting P:I ratios ranging from ~10^4^–10^8^ [65]. Such high P:I ratios measured for AAV vectors using these methods are unlikely to correspond to an overwhelming preponderance of defective AAV particles; rather, the assays that have been developed to date are insufficiently sensitive to detect all AAV vector particles that, in fact, would be measured in infectivity assays capable of detecting all competent particles. Therefore, existing AAV vector infectivity assays in general may greatly overestimate actual P:I ratios. However, they provide a relative measure of functional activity, which is useful in assessing lot-to-lot variability, vector stability and for performing comparability studies on vectors prepared using different manufacturing processes.

#### 4.5.2. Risk Assessment

A concern with having large or variable amounts of defective particles in preparations of AAV vectors intended for use in clinical studies would be similar to the risks described previously for empty capsids, *i.e.*, defective particles would provide unnecessary viral antigen, interfere with transduction by infectious particles and increase the risk for immunotoxicity. Preparations of AAV vectors with lower infectivity would require higher doses to achieve efficacy. Clinical studies using AAV vectors typically base dosing on vector genome titers rather than infectivity titers, and lot-to-lot variability in P:I ratios would render efficacy difficult to predict based on vector genome titers alone. 

#### 4.5.3. Process Optimization

The current generation of infectivity assays capable of measuring relative infectivity for specific AAV vector constructs can be used together in a matrix with other measurements of AAV vector functional activity to assess vector manufacturing steps during process optimization and for storage and handling of AAV vectors. 

### 4.6. Aggregated, Degraded, Oxidized AAV Vectors

AAV vectors may be subject to aggregation, degradation, oxidation and other physico-chemical changes during their purification and storage, which may have a deleterious effect on potency and safety. Such changes should be measured and controlled in AAV-based investigational product using appropriate strategies for the optimization of vector purification processes, formulation and storage. Such strategies are analogous to those previously developed for recombinant protein and vaccine products and will not be further considered here. 

## 5. Conclusions

In conclusion, the potential for recombinant AAV-based gene transfer to address human disease is great. A clear understanding of the provenance and risks associated with vector manufacturing process- and vector product-related impurities in AAV vectors prepared for clinical studies is needed to assure and control product purity, potency and safety. As investigational products based upon AAV continue to move through the clinical development stages from pre-clinical proof-of-concept, Good Laboratory Practice (GLP) toxicology studies, Phase I through III clinical studies and, finally, to new product licensure, a clear understanding of the risks associated with impurities that may be present, their minimization and control and the implementation of optimized methods for manufacturing AAV vectors along with validated analytical controls are critical to ensure that the promise of these promising new therapeutic products is fully achieved. 
